# Multivariate Higher-Order IRT Model and MCMC Algorithm for Linking Individual Participant Data From Multiple Studies

**DOI:** 10.3389/fpsyg.2019.01328

**Published:** 2019-06-12

**Authors:** Eun-Young Mun, Yan Huo, Helene R. White, Sumihiro Suzuki, Jimmy de la Torre

**Affiliations:** ^1^University of North Texas Health Science Center, Fort Worth, TX, United States; ^2^Educational Testing Service, Princeton, NJ, United States; ^3^Rutgers University Center of Alcohol Studies, Piscataway, NJ, United States; ^4^The University of Hong Kong, Pokfulam, Hong Kong

**Keywords:** higher-order IRT, multivariate IRT, multi-group IRT, Bayesian estimation, individual participant data, meta-analysis

## Abstract

Many clinical and psychological constructs are conceptualized to have multivariate higher-order constructs that give rise to multidimensional lower-order traits. Although recent measurement models and computing algorithms can accommodate item response data with a higher-order structure, there are few measurement models and computing techniques that can be employed in the context of complex research synthesis, such as meta-analysis of individual participant data or integrative data analysis. The current study was aimed at modeling complex item responses that can arise when underlying domain-specific, lower-order traits are hierarchically related to multiple higher-order traits for individual participant data from multiple studies. We formulated a multi-group, multivariate higher-order item response theory (HO-IRT) model from a Bayesian perspective and developed a new Markov chain Monte Carlo (MCMC) algorithm to simultaneously estimate the (a) structural parameters of the first- and second-order latent traits across multiple groups and (b) item parameters of the model. Results from a simulation study support the feasibility of the MCMC algorithm. From the analysis of real data, we found that a bivariate HO-IRT model with different correlation/covariance structures for different studies fit the data best, compared to a univariate HO-IRT model or other alternate models with unreasonable assumptions (i.e., the same means and covariances across studies). Although more work is needed to further develop the method and to disseminate it, the multi-group multivariate HO-IRT model holds promise to derive a common metric for individual participant data from multiple studies in research synthesis studies for robust inference and for new discoveries.

## Introduction

Item response theory (IRT; Hambleton and Swaminathan, 1985; Van der Linden and Hambleton, 1997; Embretson and Reise, 2000) is a modern psychometric theory that provides a statistical modeling framework for expressing observed item responses as a function of latent (unobserved) traits (e.g., abilities, attributes, psychological constructs). IRT has been extensively used in educational testing and measurement, psychological assessment, and health outcomes measurement (Bartolucci et al., 2015; Gibbons et al., 2016). The present study draws from the IRT literature to retrospectively establish a common metric across independently conducted studies with different items and response options. Having a common metric or understanding of how scores from different studies can be made comparable is important in comparative effectiveness research. However, in part because it is difficult to observe and quantify unobserved latent traits, different measures for assessing the same trait have proliferated in the literature. For example, more than 280 different depression scales have been developed and used in the past century (Fried and Flake, 2018). Any measurement differences between studies ultimately need to be taken into account when interpreting effect sizes. However, it is virtually impossible to empirically tease apart the sources of between-study heterogeneity because study-level differences are often confounded with one another (e.g., some of the trials aimed at seriously ill patients may have used outcome measures intended to capture more serious symptoms). Consequently, any resulting interpretation of treatment effect sizes obtained from studies that used different outcome measures would be subjective and difficult to verify. Therefore, combining effect sizes from different outcome measures in meta-analysis has long been discouraged (Hedges and Olkin, 1985).

Prospectively, it is possible to link different items from multiple surveys and questionnaires by testing them using the single group design or the anchor test design (Streiner et al., 2015; e.g., Choi et al., 2014 for depression). Other non-IRT methods also exist to link items. However, the existing approaches have limited utility when there is no established “standard” metric to compare measures against, and when the number of utilized measures is high because it would be impractical to study all measures. More important, these approaches require preplanning to establish measurement equivalence in a separate investigation. Therefore, there is a need for new methods and approaches that directly address the measurement challenge for meta-analysis studies combining individual participant data (IPD) or for integrative data analysis (IDA; Curran and Hussong, 2009).

## Advances in IRT and Challenges for IPD Meta-Analysis

Traditionally, IRT involves a unidimensional underlying trait, denoted as θ. The two most basic and common unidimensional IRT models are the Rasch model and the two-parameter logistic (2PL) model. There are two major assumptions involved in IRT—unidimensionality and local independence. The unidimensionality assumption of IRT requires that a single latent dimension θ substantially accounts for the way participants respond to items. The local independence assumption describes that items should be conditionally independent given θ. The IRT models for binary item response can be extended to accommodate polytomous item response in a number of related models (Bacci et al., 2014).

Further, as an extension of the unidimensional IRT model, multidimensional IRT (MIRT) models can accommodate joint modeling of multiple dimensions, typically expressed as **θ**. The bifactor IRT model (Gibbons and Hedeker, 1992) is one of the most well-known MIRT models. Although MIRT models have greater generality and may provide greater flexibility in real data applications, they are associated with more complexity in terms of both model parameterization and estimation, compared to traditional unidimensional IRT models. MIRT models can also be modified to accommodate multidimensional discrete latent traits (instead of continuous latent traits **θ)** so that the lower-order latent traits are construed as latent classes, which can be seen as a latent class IRT model. A number of studies pertaining to multidimensional latent class IRT models have become available in recent years. For example, Bartolucci (2007) proposed a class of MIRT models that measures discrete latent traits in the context of binary items. More recently, Gnaldi et al. (2016) extended the model to incorporate item response data nested within students that are nested in schools.

More broadly, there are a number of new IRT models and factor analysis (FA) models and software programs that can account for multidimensional data with a higher-order structure (e.g., Sheng and Wikle, 2008; de la Torre and Song, 2009; de la Torre and Hong, 2010; Huang and Wang, 2013, 2014; Huang et al., 2013; Rijmen et al., 2014). Other recent models can also accommodate respondents' or testlets' effects in multilevel, multi-group, and/or mixture IRT models (e.g., van der Linden, 2007; Klein Entink et al., 2009; Cho and Cohen, 2010; Azevedo et al., 2015; Camilli and Fox, 2015; Fox and Marianti, 2016; Schmidt et al., 2016). In addition, Bayesian estimation is possible using general purpose software programs, such as Mplus (with Bayes estimator; Muthén and Muthén, 1998-2018), Stan (Bürkner, 2017), or WinBUGS (Lunn et al., 2000).

Despite the advances noted above, it is challenging to apply existing IRT or FA models and associated computing algorithms to item-level IPD obtained from independently conducted studies (see Huo et al., 2015; Mun et al., 2015). Some of the major challenges include study-level missing data, limited item overlap across studies, and other between-study differences (Hussong et al., 2013; Mun et al., 2015). These challenges associated with analyzing IPD from multiple sources have been discussed extensively elsewhere (e.g., Hussong et al., 2013; Mun et al., 2015; Brincks et al., 2018; Siddique et al., 2018). In spite of the challenges, it is of critical importance that commensurate measures for participants from multiple studies be established as the first major step toward ensuring the same data interpretation across studies for IDA or meta-analysis of IPD.

## Multivariate HO-IRT Model for Item Response Data From Multiple Studies

We focus on psychological constructs with a multivariate higher-order structure, which give rise to multidimensional lower-order traits in the present study. A multivariate higher-order item response theory (HO-IRT) model is developed to estimate trait scores of participants from multiple studies and tested using a Markov chain Monte Carlo (MCMC) estimation approach. We use the term “multidimensional” to indicate that multiple, possibly related traits give rise to observed item response data. A “hierarchical structure” or “higher-order structure” is said to exist when multiple lower-order traits can be expressed as function of an overall, higher-order trait. When there are two or more higher-order traits, a “multivariate” higher-order structure exists.

It has been noted that a lack of new methods and application examples to address the challenges of analyzing data from multiple studies has hindered the broader adoption of IDA by applied researchers despite its promise (Curran et al., 2017). On one hand, it is difficult to fully grasp the unique challenges of analyzing IPD from multiple studies and to be motivated to develop new methods and algorithms, yet; on the other hand, without the algorithms designed for IPD obtained from multiple studies, there would be no reports to share, resulting in a “catch 22” situation. If there were more options available, applied researchers could choose more appropriate analytic approaches to meet their needs in the context of subsequent analyses, which would be more preferable than either invoking unreasonable data assumptions or not considering research synthesis even when it is feasible (Mun and Ray, 2018).

In sum, the present study is aimed at addressing the aforementioned gaps in available methods by developing a multivariate HO-IRT model and associated computing algorithm. Our rationale for the development of the multivariate HO-IRT model is two-fold. First, many clinical and psychological constructs have been conceptualized to have multivariate higher-order constructs that give rise to multidimensional lower-order traits; yet most of the available measurement models for the purpose of analyzing existing data from multiple studies are unidimensional and non-hierarchical. Second, the multivariate HO-IRT model may be appealing for certain research applications because it estimates multiple trait scores at the higher-order, as well as lower-order, levels, thereby achieving data reduction. Having more options in our methodological tool box can empower researchers in the field of psychology in their pursuit of fully maximizing available data for new discoveries. In the following sections, we describe the multivariate HO-IRT model, MCMC estimation, results from a simulation study, and application results from a motivating data example.

## Multivariate HO-IRT Model: Model Specification

**Figure 1:**
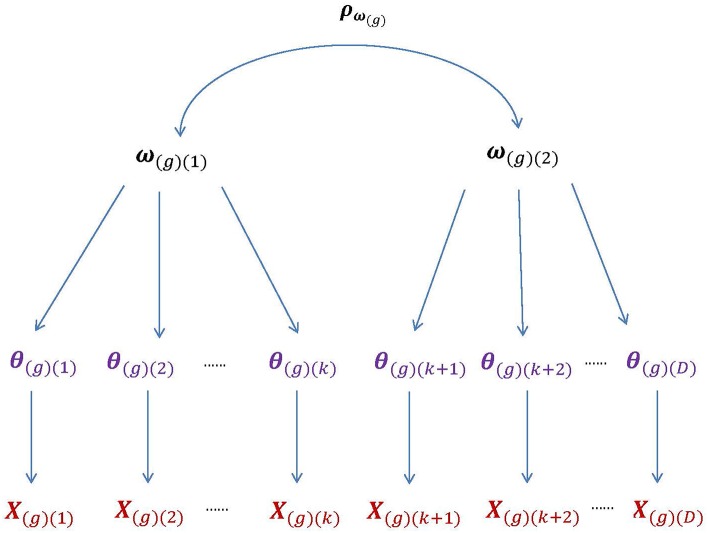
The higher-order structure of the bivariate HO-IRT model for multiple groups. The second-order latent trait for group *g*, **ω**_(*g*)_, is bivariate with the correlation matrix **ρ**_**ω**_*(g)*__, whereas the first-order latent trait, **θ**_(*g*)_, is *D*-dimensional. The first higher-order component, **ω**_(*g*)__(1)_, are related to the first *k* components (i.e., dimensions) of **θ**, whereas the second higher-order component, **ω**_(*g*)__(2)_, to the remaining (*D* − *k*) components. Further, the components of the first-order trait **θ**_(*g*)_ are assumed to be independent conditional on the second-order traits **ω**_(*g*)_.

In univariate HO-IRT models (de la Torre and Song, 2009; de la Torre and Hong, 2010), θ_(*d*)_ is the domain-specific, first-order latent trait for the *d*th domain with *d* = 1, 2, …, *D*. The *D* dimensions of **θ** can be linked to a single overall latent trait ω via a weighted linear function: θ_*i*(*d*)_ = λ_(*d*)_ω_*i*_+ε_*i*(*d*)_, where the subscript [*i*] indexes participant, λ_(*d*)_ is the regression coefficient, and ε_*i*(*d*)_ is the residual term conditioned on ω_*i*_. However, **θ** may not be sufficiently captured by a single overall latent trait. In such a case, we need to extend the univariate HO-IRT model to accommodate a vector of higher-order latent traits (i.e., **ω**) when describing relationships between first- and second-order latent traits. Figure 1 graphically shows the structure of the multivariate HO-IRT model for multiple groups with the subscript *g* indicating group (or study).

In this paper, we use the hierarchical, multi-unidimensional two-parameter logistic item response theory (2PL-MUIRT) model as the item response function (Huo et al., 2015), where each item individually measures a single dimension and all items collectively measure the *D* dimensions of **θ**. Specifically,

$$\begin{array}{l} P\left(X_{i\left(g\right)j\left(d\right)}=1|\theta_{i\left(g\right)\left(d\right)},\;\alpha_{j\left(d\right)},\;\beta_{j\left(d\right)}\right)\\ =\frac{\exp\left[\alpha_{j\left(d\right)}\left(\theta_{i\left(g\right)\left(d\right)}-\beta_{j\left(d\right)}\right)\right]}{1+\text{exp}\left[\alpha_{j\left(d\right)}\left(\theta_{i\left(g\right)\left(d\right)}-\beta_{j\left(d\right)}\right)\right]}, \end{array}$$

where *X*_*i*(*g*)*j*(*d*)_ is the response of respondent *i* in group *g* to the *j*th item of the *d*th dimension; θ_*i*(*g*)__(*d*)_ is the *d*th component of vector **θ**_*ig*_ = {θ_*i*(*g*)__(*d*)_}; α_*j*(*d*)_ and β_*j*(*d*)_ are the discrimination and difficulty parameters, respectively, of the *j*th item of the *d*th dimension; *j*(*d*) = 1(*d*), 2(*d*), …, *J*(*d*); and $\overset{D}{\underset{d=1}{\sum }}J\left(d\right)=J$. All item parameters are assumed to be the same across the *g* = 1, 2, …, *G* groups. The likelihood of the data matrix **X**_*g*_ (*g* = 1, 2, …, *G*) is given by

$$\begin{array}{l} L\left(X_{g}|\theta_{g},\;\alpha ,\;\beta \right)\\ \;=\prod_{d=1}^{D}\prod_{i=1}^{N}\prod_{j\left(d\right)=1}^{J(d)}P{\left(X_{i\left(g\right)j\left(d\right)}=1|\theta_{i\left(g\right)\left(d\right)},\;\alpha_{j\left(d\right)},\;\beta_{j\left(d\right)}\right)}^{X_{i\left(g\right)j\left(d\right)}}\\ \;\times {\left[1-P\left(X_{i\left(g\right)j\left(d\right)}=1|\theta_{i\left(g\right)\left(d\right)},\;\alpha_{j\left(d\right)},\;\beta_{j\left(d\right)}\right)\right]}^{1-X_{i\left(g\right)j\left(d\right)}}. \end{array}$$

**Figure 2:**
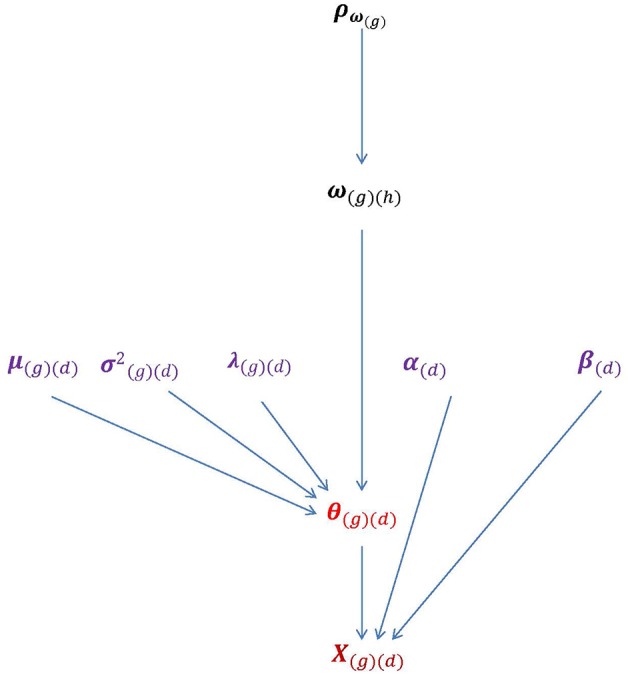
The parameters of the bivariate HO-IRT model for multiple groups. Lower-order trait scores **θ**_(*g*)__(*d*)_ can be seen as a direct function of higher-order latent traits **ω**_(*g*)__(*h*)_ and characterized by regression coefficients **λ**_(*g*)__(*d*)_ that relate **θ**_(*g*)__(*d*)_ to **ω**_(*g*)__(*h*)_ as well as by the mean vector and covariance matrices **μ**_(*g*)__(*d*)_ and $\sigma_{\left(g\right)\left(d\right)}^{2}$. Item parameters **α**_*j*(*d*)_ and **β**_*j*(*d*)_ are also displayed.

To take multiple groups into account, the following function can be used to connect **θ** to **ω**: θ_*i*(*g*)__(*d*)_ = λ_(*g*)__(*d*)_ω_*i*(*g*)__(*h*)_+ε_*i*(*g*)__(*d*)_. The subscript *h* = 1, 2, …, *H* denotes the *h*th dimension of **ω**. When *H* takes the value of 1, 2, or 3 or more, the multivariate HO-IRT model can be described specifically as univariate, bivariate, and multivariate models, respectively. Figure 2 shows the essential parameters of the multivariate HO-IRT model to be estimated from a Bayesian perspective. The proposed multivariate HO-IRT model shares the same item response function with the hierarchical 2PL-MUIRT model but differs in its approach to estimating covariance structures among first-order latent traits across groups. In the hierarchical 2PL-MUIRT model, the correlation or covariance structures of the first-order latent traits are *directly* estimated as unknown parameters. In contrast, in the proposed multivariate HO-IRT model, the correlation or covariance structures of first-order latent traits are estimated *indirectly* as a function of the estimated regression coefficients relating first-order traits to second-order traits and the variances of the first-order latent traits.

## Markov Chain Monte Carlo Estimation

### Prior, Posterior, and Conditional Distributions

We use the hierarchical Bayesian formulation with the following prior distributions:

$$\begin{array}{l} \mu_{\left(g\right)\left(d\right)}\sim MVN\left(\mu_{H},\;\Sigma_{H}\right), \end{array}$$

where **μ**_*H*_ was set to **0** and **Σ_*H*_** was a correlation matrix with the off-diagonal element set to 0.5;

$$\begin{array}{l} \;\sigma_{\left(g)(d\right)}^{2}\sim U\left(0,\;1\right);\\ \;\lambda_{\left(g)(d\right)}\sim U\left(-1,\;1\right);\\ \;\omega_{i(g)}\sim MVN\left(\left(\begin{array}{c} 0\\ 0 \end{array}\right),\;\left(\begin{array}{c} 1\;0\\ 0\;1 \end{array}\right)\right);\\ \;\theta_{i\left(g\right)\left(d\right)}\sim N\left(\mu_{\left(g\right)\left(d\right)},\;\sigma_{\left(g\right)\left(d\right)}^{2}\right);\\ \alpha_{j(d)}\sim 4Beta\left(\upsilon_{0\alpha },\;\omega_{0\alpha },\;a_{0\alpha },\;b_{0\alpha }\right);\;and\\ \;\beta_{j(d)}\sim 4Beta\left(\upsilon_{0\beta },\;\omega_{0\beta },\;a_{0\beta },\;b_{0\beta }\right), \end{array}$$

where 4*Beta* indicates the four-parameter beta distribution with υ and ω as shape parameters and *a* and *b* as the location parameters defining the support of the distribution. **ρ**_**ω**_(*g*)__ needs to be transformed from **Σ**_**ω**_(*g*)__, which we describe later in this section. Here,

$$\begin{array}{l} \Sigma_{\omega_{\left(g\right)}}\sim Inverse\;Wishart\left(\nu_{0}=D+2,\;\Lambda_{0}^{-1}=\left(\begin{array}{c} 1\;0.2\\ 0.2\;1 \end{array}\right)\right). \end{array}$$

The priors were chosen based on previous studies (e.g., de la Torre and Patz, 2005; de la Torre and Song, 2009; Huo et al., 2015) and adapted to meet the goals of the current study.

The joint posterior distribution of interest is as follows:

$$\begin{array}{l} P\left(\theta ,\;\omega ,\;\mu ,\;\sigma^{2},\;\lambda ,\;\rho_{\omega },\;\alpha ,\;\beta |X\right)\propto [\prod_{g=1}^{G}L\left(X_{\left(g\right)}|\theta_{\left(g\right)},\;\alpha ,\;\beta \right)\\ P\left(\theta_{\left(g\right)}\right)P\left(\omega_{\left(g\right)}\right)\;P\left(\mu_{\left(g\right)}\right)P\left(\sigma^{2}{}_{\left(g\right)}\right)P\left(\lambda_{\left(g\right)}\right)P\left(\rho_{\omega_{\left(g\right)}}\right)]\\ \times P\left(\alpha \right)P\left(\beta \right), \end{array}$$

which cannot be fully simplified into an explicit distribution from which samples can be drawn directly. Therefore, we decompose the joint posterior distribution into several full conditional distributions for samples to be drawn more easily by using either the direct sampling approach (i.e., Gibbs sampling; Casella and George, 1992) or the indirect sampling approach (i.e., Metropolis-Hasting [M-H] algorithm; e.g., Chib and Greenberg, 1995). The full conditional distribution of **θ**_(*g*)_ is

$$\begin{array}{l} P\left(\theta_{\left(g\right)}|{\text{X}}_{\left(g\right)},\omega_{\left(g\right)},\;\mu_{\left(g\right)},\;{\sigma^{2}}_{\left(g\right)},\;\lambda_{\left(g\right)},\;\alpha ,\;\beta \right)\\ \propto L\left({\text{X}}_{\left(g\right)}|\theta_{\left(g\right)},\;\alpha ,\;\beta \right)P\left(\theta_{\left(g\right)}|\omega_{\left(g\right)},\;\mu_{\left(g\right)},\;{\sigma^{2}}_{\left(g\right)},\;\lambda_{\left(g\right)}\right). \end{array}$$

The full conditional distribution of **ω**_(*g*)_ is

$$\begin{array}{l} P\left(\omega_{\left(g\right)}|{\text{X}}_{\left(g\right)},\theta_{\left(g\right)},\;\mu_{\left(g\right)},\;{\sigma^{2}}_{\left(g\right)},\;\lambda_{\left(g\right)},\;\alpha ,\;\beta \right)\\ \propto P\left(\omega_{\left(\text{g}\right)}\right)P\left(\theta_{\left(g\right)}|\omega_{\left(g\right)},\;\mu_{\left(g\right)},\;{\sigma^{2}}_{\left(g\right)},\;\lambda_{\left(g\right)}\right). \end{array}$$

The full conditional distribution of **μ**_(*g*)_ is *MVN*(**μ**_1(*g*)_, **Σ**_1(*g*)_), where the parameters can explicitly be expressed as

$$\begin{array}{lll} \mu_{1\left(g\right)} & = & {\left(\Lambda_{0\left(g\right)}^{-1}+N_{\left(g\right)}\Sigma_{\left(g\right)}^{-1}\right)}^{-1}\left({\Lambda_{0\left(g\right)}^{-1}\mu }_{0\left(g\right)}+N_{\left(g\right)}\Sigma_{\left(g\right)}^{-1}{\bar{\theta }}_{\left(g\right)}\right);\\ \Sigma_{1\left(g\right)} & = & {\left(\Lambda_{0\left(g\right)}^{-1}+N_{\left(g\right)}\Sigma_{\left(g\right)}^{-1}\right)}^{-1}. \end{array}$$

Samples can be drawn directly from this distribution using Gibbs sampling. The full conditional distribution of ${\sigma^{2}}_{\left(g\right)}$ is

$$\begin{array}{l} P\left(\sigma_{\left(g\right)}^{2}|{\text{X}}_{\left(g\right)},{\theta_{\left(g\right)},\;\omega }_{\left(g\right)},\;\mu_{\left(g\right)},\;\lambda_{\left(g\right)},\;\alpha ,\;\beta \right)\\ \propto P\left(\sigma_{\left(g\right)}^{2}\right)P\left(\theta_{\left(g\right)}|\omega_{\left(g\right)},\;\mu_{\left(g\right)},\;{\sigma^{2}}_{\left(g\right)},\;\lambda_{\left(g\right)}\right), \end{array}$$

and similarly the full conditional distribution of **λ**_(***g***)_ is

$$\begin{array}{l} P\left(\lambda_{\left(g\right)}|{\text{X}}_{\left(g\right)},\theta_{\left(g\right)},\omega_{\left(g\right)},\;\mu_{\left(g\right)},\;{\sigma^{2}}_{\left(g\right)},\;\alpha ,\;\beta \right)\\ \propto P\left(\lambda_{\left(g\right)}\right)P\left(\theta_{\left(g\right)}|\omega_{\left(g\right)},\;\mu_{\left(g\right)},\;{\sigma^{2}}_{\left(g\right)},\;\lambda_{\left(g\right)}\right). \end{array}$$

As previously noted, **ρ**_**ω**_(*g*)__ needs to be drawn from **Σ**_**ω**_(*g*)__, which has a full conditional distribution of

$$\begin{array}{l} P\left(\Sigma_{\omega_{\left(g\right)}}|\omega_{g}\right)\propto P\left(\Sigma_{\omega_{\left(g\right)}}\right)P\left(\omega_{g}|\Sigma_{\omega_{\left(g\right)}}\right), \end{array}$$

and can be directly sampled using Gibbs sampling. To draw **ρ**_**ω**_(*g*)__, first, samples are drawn from the conditional distribution of **Σ**_**ω**_(*g*)__, as in the Inverse Wishart distribution (see Gelman et al., 2004), and the sampled **Σ**_**ω**_(*g*)__ is then transformed into the corresponding provisional **ρ**_**ω**_(*g*)__, which is evaluated by the M-H acceptance criteria. This method was originally developed by Liu (2008) and Liu and Daniels (2006), and utilized in Huo et al. (2015). Finally, the full conditional distribution of the item parameters is

$$\begin{array}{l} P\left(\alpha ,\;\beta |\text{X},\;\theta \right)\\ \;\propto \overset{N}{\underset{i=1}{\prod }}\overset{G}{\underset{g=1}{\prod }}\overset{D}{\underset{d=1}{\prod }}\overset{J\left(d\right)}{\underset{j\left(d\right)=1}{\prod }}L\left(X_{i\left(g\right)j\left(d\right)}|\theta_{i\left(g\right)\left(d\right)},\;\alpha_{j\left(d\right)},\;\beta_{j\left(d\right)}\right)\\ \;\times P\left(\alpha_{j\left(d\right)}\right)P\left(\beta_{j\left(d\right)}\right). \end{array}$$

For the full conditional distributions for $\theta_{\left(g\right)},\;\omega_{\left(g\right)},\;{\sigma^{2}}_{\left(g\right)},\;$and **λ**_(*g*)_, the M-H algorithm can be used to indirectly draw samples from the corresponding distributions. The multivariate HO-IRT model with multiple groups formulated above is a full model containing the multivariate higher-order latent traits for multiple groups. Details of the MCMC algorithms are shown in the Appendix.

### Estimation Indeterminacy

We note two unique challenges to address when estimating a multivariate HO-IRT model for multiple groups. The first challenge involves addressing estimation indeterminacy. We set constraints on the latent distributions by selecting an anchor group, setting its mean to be **0**, and constraining its covariance matrix to be equivalent to a correlation matrix. Along with the estimation of the correlation matrix for the anchor group, the mean vectors and covariance matrices of the remaining groups are directly estimated by modifying the relevant MCMC algorithms (Liu and Daniels, 2006; Liu, 2008), and adapting them to the multivariate HO-IRT model. Alternatively, constraints can be imposed on item parameters (e.g., fixing one discrimination parameter to 1 and one difficulty parameter to 0; e.g., Fox and Glas, 2001) without setting any particular anchor group. Although both options address estimation indeterminacy, we chose the first, which is more consistent with the IRT tradition of constraining the latent distributions (i.e., structural parameters) rather than the item parameters.

The second challenge is in constructing the correlation/covariance structures of **θ**|**ω**. For each group, conditioned on **ω**, **θ**s are independent from one another. The conditional variance of **θ**_(*d*)_|**ω** is $\sigma_{\left(d\right)}^{2}\left(1-\lambda_{\left(d\right)}^{2}\right)$. However, the **θ**s are not marginally independent across dimensions. The elements of the correlation or covariance matrices can be derived from $\sigma_{\left(d\right)}^{2}$ for θ_(*d*)_. Given that the covariance matrix of the anchor group is constrained, the covariance matrix θ_(*d*)_ for the anchor group is equivalent to the correlation matrix because $\sigma_{\left(d\right)}^{2}=1$. Therefore, for any two θs (e.g., θ_*d*_ and $\theta_{d^{\prime }}$, *d*≠*d*′) sharing the same higher-order ω, their covariances can be estimated as σ_(d)_σ_(d′)_λ_(d)_λ_(d′)_. If two θs do not share the same ω, the covariances can be estimated as σ_(d)_σ_(d′)_λ_(d)_λ_(d′)_ρ_ω_.

## Simulation Study

### Simulation Design

A simulation study was conducted to evaluate the feasibility of the MCMC algorithms for the bivariate HO-IRT model with multiple groups. In this simulation study we examined the bivariate HO-IRT model with multiple groups in the saturated form, that is, different means and covariance structures for bivariate second-order latent traits across groups. In other words, the feasibility of the most comprehensive MCMC algorithm for a saturated (full) model was examined because any reduced models are special cases of the saturated model with constraints. More specifically, for the simulation design, three groups with each having 1,000 participants were specified. The second-order latent trait, **ω**_(*g*)_, for the three groups was set to be identical. Specifically, the underlying distribution for the second-order latent trait was bivariate normal with **μ** = **0**, **Σ** = **R**, and ρ = 0.5. The first-order latent traits θ_*i*(*g*)__(*d*)_ were generated from $MVN\left(\mu_{\left(g\right)\left(d\right)}+\lambda_{\left(g\right)\left(d\right)}\sigma_{\left(g\right)\left(d\right)}\omega_{i\left(g\right)\left(d\right)},\;\sigma_{\left(g\right)\left(d\right)}^{2}\left(1-\lambda_{\left(g\right)\left(d\right)}^{2}\right)\right)$. The true parameters of **μ**, **λ**, **σ**^2^, and covariance matrices are presented in Table 1. Note that the first group was designated as the anchor group with a zero mean vector and a correlation matrix.

**Table 1:** True parameter values of **μ**, **λ**, and **σ**^2^ for the first-order traits **θ**_(*d*)_ in the simulation study.

**Group**	**λ**	**μ**	**σ^2^**	**Covariance**
1	$\left(\begin{array}{c} 0.837\\ 0.837\\ 0.837\\ 0.837\\ 0.837 \end{array}\right)$	$\left(\begin{array}{c} 0\\ 0\\ 0\\ 0\\ 0 \end{array}\right)$	$\left(\begin{array}{c} 1\\ 1\\ 1\\ 1\\ 1 \end{array}\right)$	$\left(\begin{array}{ccccc} 1\; & \; & \; & \; & \;\\ 0.7 & 1 & \; & \; & \;\\ 0.7 & 0.7 & 1 & \; & \;\\ 0.35 & 0.35 & 0.35 & 1 & \;\\ 0.35 & 0.35 & 0.35 & 0.7 & 1 \end{array}\right)$
2	$\left(\begin{array}{c} 0.837\\ 0.837\\ 0.837\\ 0.837\\ 0.837 \end{array}\right)$	$\left(\begin{array}{c} 0.3\\ 0.4\\ 0.5\\ 0.6\\ 0.7 \end{array}\right)$	$\left(\begin{array}{c} 0.75\\ 0.75\\ 0.75\\ 0.75\\ 0.75 \end{array}\right)$	$\left(\begin{array}{ccccc} 0.75 & \; & \; & \; & \;\\ 0.53 & 0.75 & \; & \; & \;\\ 0.53 & 0.53 & 0.75 & \; & \;\\ 0.26 & 0.26 & 0.26 & 0.75 & \;\\ 0.26 & 0.26 & 0.26 & 0.53 & 0.75 \end{array}\right)$
3	$\left(\begin{array}{c} 0.837\\ 0.837\\ 0.837\\ 0.837\\ 0.837 \end{array}\right)$	$\left(\begin{array}{c} -0.3\\ -0.4\\ -0.5\\ -0.6\\ -0.7 \end{array}\right)$	$\left(\begin{array}{c} 1.25\\ 1.25\\ 1.25\\ 1.25\\ 1.25 \end{array}\right)$	$\left(\begin{array}{ccccc} 1.25 & \; & \; & \; & \;\\ 0.88 & 1.25 & \; & \; & \;\\ 0.88 & 0.88 & 1.25 & \; & \;\\ 0.44 & 0.44 & 0.44 & 1.25 & \;\\ 0.44 & 0.44 & 0.44 & 0.88 & 1.25 \end{array}\right)$

*Underlying distribution for the second-order latent traits is bivariate normal with **μ** = **0**, **Σ** = **R**, and ρ = 0.5*.

The complete item responses were generated based on **θ**s and item parameters. Thirty items were randomly drawn from 80 2PL item parameters in an item pool. The item parameters were constructed based on our prior experience of analyzing similar data. Their discrimination and difficulty parameters are presented in Table 2. To ensure that item quality is identical across dimensions, these 30 items were repeated five times and used in the simulation phase as a 150-item test set, measuring five correlated traits. All three groups (a total sample size of 3,000) had complete responses on all 150 items. A total of 25 replication data sets were generated and analyzed. All estimation codes were written and implemented using Ox, an object oriented programming language (Doornik, 2009), and can be made available to interested readers upon request.

**Table 2:** Item parameters used in the simulation study (First 30 items).

	**Discrimination**	**Difficulty**		**Discrimination**	**Difficulty**
**Item**	**(α)**	**(β)**	**Item**	**(α)**	**(β)**
1	1.288	0.193	16	1.554	0.693
2	1.320	−0.080	17	1.390	1.076
3	1.260	0.881	18	0.930	−0.668
4	1.092	1.300	19	0.906	−0.028
5	1.120	0.164	20	1.366	1.852
6	0.995	1.096	21	1.258	1.821
7	1.010	0.562	22	0.919	1.797
8	1.366	1.488	23	0.944	1.751
9	1.110	−1.351	24	1.253	−0.654
10	0.956	1.557	25	0.910	−1.013
11	1.050	0.134	26	0.977	−0.942
12	0.937	−0.408	27	0.974	−0.244
13	0.682	1.503	28	1.231	−0.604
14	1.125	1.504	29	0.780	−1.236
15	1.105	1.746	30	1.099	−1.162

### Simulation Results

Four MCMC chains were simultaneously run to monitor their convergence. Each chain had 25,000 iterations and the first 10,000 iterations were discarded as burn-in. The Gelman–Rubin (G–R) diagnostic statistics (Gelman and Rubin, 1992) across all parameters were <1.1 (when tested with a single replication and one chain), suggesting that convergence was achieved. Table 3 presents the estimation results. The MCMC estimates of the means were almost identical to their true values. The computed standard errors (*SE*s) were small and generally comparable across different **μ**s. The estimates of **λ***s* for the three groups ranged from 0.827 to 0.840, which indicates a fairly good estimation recovery of their true value, 0.837. The *SE*s were generally small and consistent across dimensions and groups. The estimates of **σ**^2^s were also quite close to their respective true values, and the estimated correlations between the two second-order traits were very close to the true correlation (0.5) in all three groups. Table 4 presents the bias and RMSE of the discrimination and difficulty parameter estimates of the 150 items as well as for the entire test. Overall, the magnitudes of the bias and RMSE of the discrimination and difficulty parameter estimates were very small (i.e., maximum absolute bias and RMSE of 0.008 and 0.014, respectively), indicating that these item parameters were accurately recovered.

**Table 3:** Estimated parameters and *SE*s of the simulated bivariate HO-IRT model for three groups (data averaged across 25 replications).

	**Group 1**		**Group 2**		**Group 3**	
**ω**	${\hat{\rho }}_{\omega_{1}}$	$SE_{{\hat{\rho }}_{\omega_{1}}}$	${\hat{\rho }}_{\omega_{2}}$	$SE_{{\hat{\rho }}_{\omega_{2}}}$	${\hat{\rho }}_{\omega_{3}}$	$SE_{{\hat{\rho }}_{\omega_{3}}}$
	0.500	0.013	0.496	0.014	0.497	0.009
**θ**	${\hat{\lambda }}_{1}$	$SE_{{\hat{\lambda }}_{1}}$	${\hat{\lambda }}_{2}$	$SE_{{\hat{\lambda }}_{2}}$	${\hat{\lambda }}_{3}$	$SE_{{\hat{\lambda }}_{3}}$
1	0.831	0.010	0.835	0.012	0.833	0.014
2	0.838	0.012	0.833	0.012	0.840	0.010
3	0.833	0.010	0.831	0.014	0.836	0.011
4	0.827	0.015	0.837	0.012	0.834	0.019
5	0.838	0.012	0.834	0.017	0.836	0.015
**θ**			${\hat{\mu }}_{2}$	$SE_{{\hat{\mu }}_{2}}$	${\hat{\mu }}_{3}$	$SE_{{\hat{\mu }}_{3}}$
1	NA	NA	0.300	0.014	−0.305	0.016
2	NA	NA	0.403	0.016	−0.404	0.021
3	NA	NA	0.505	0.015	−0.498	0.021
4	NA	NA	0.605	0.013	−0.600	0.021
5	NA	NA	0.704	0.013	−0.702	0.019
**θ**			${\hat{\sigma }}_{2}^{2}$	$SE_{{\hat{\sigma }}_{2}^{2}}$	${\hat{\sigma }}_{3}^{2}$	$SE_{{\hat{\sigma }}_{3}^{2}}$
1	NA	NA	0.759	0.027	1.283	0.047
2	NA	NA	0.768	0.027	1.278	0.051
3	NA	NA	0.757	0.025	1.273	0.044
4	NA	NA	0.742	0.026	1.270	0.055
5	NA	NA	0.758	0.027	1.267	0.042

*True values can be seen in Table 1. NA, due to the constraints set for the anchor group*.

**Table 4:** Bias and RMSE of the discrimination and difficulty parameter estimates of the 150 items.

	**Bias**		**RMSE**	
**θ**	**Discrimination**	**Difficulty**	**Discrimination**	**Difficulty**
	**(α)**	**(β)**	**(α)**	**(β)**
1	−0.002	0.003	0.013	0.013
2	−0.008	0.004	0.014	0.014
3	−0.005	0.005	0.012	0.011
4	0.003	0.005	0.013	0.009
5	−0.001	0.004	0.010	0.012
Overall	−0.002	0.004	0.012	0.012

**Figure 3:**
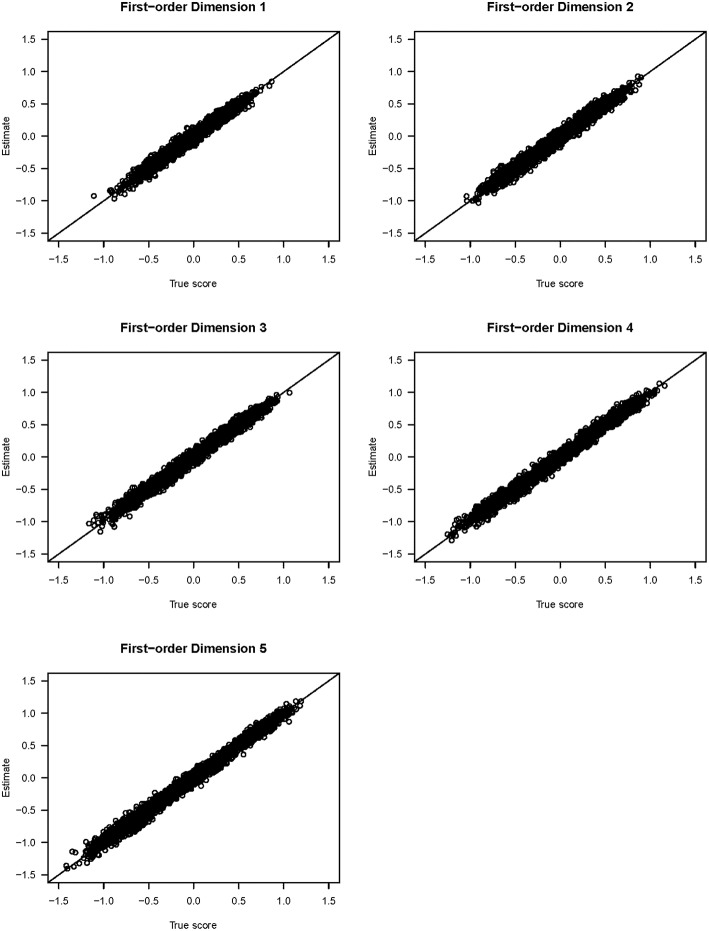
Scatter plots of the true and estimated first-order latent trait scores from the simulation study.

**Figure 4:**
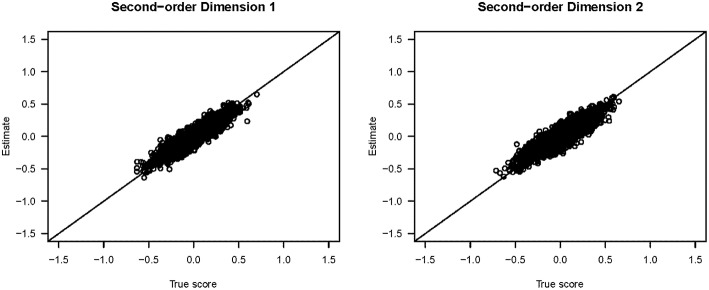
Scatter plots of the true and estimated second-order latent trait scores from the simulation study.

Figure 3 shows the scatter plots of the true and estimated first-order latent trait scores from 3,000 respondents (from all three groups) averaged across 25 replications. All five plots show that the true and estimated trait scores were highly correlated (Pearson's *r* ≥ 0.985). Similarly, Figure 4 shows the scatter plots of the true and estimated second-order latent trait scores for each of the two second-order dimensions. The two plots in Figure 4 show that the true and estimated trait scores at this second-order level were highly correlated (Pearson's *r* = 0.914 and 0.884, respectively, for the two second-order trait dimensions).

Although the simulation study illustrated the MCMC algorithms for the bivariate HO-IRT model in the saturated form, the MCMC algorithms can flexibly be adapted to fit reduced (i.e., simpler) models. For example, a reduced model may be a bivariate HO-IRT model with multiple groups with different means and a common covariance structure across groups. This reduced model may be needed for studies with small samples or sparse data, because the estimation of different covariance structures across groups may result in unreliable estimates under challenging data situations. With a common covariance structure assumed among multiple groups, it is straightforward to estimate the reduced models. In this case, only one set of the common **λ** and **σ**^2^ needs to be specified in the model. Moreover, additional model constraints can be imposed to the anchor group to avoid estimation indeterminacy, which renders the common covariance matrix being equivalent to the common correlation matrix. The implied correlation between any two θs sharing the same second-order ω simplifies to λ_(d)_ × λ_(d′)_, and between any two θs stemming from two different ωs to λ_(d)_ × λ_(d′)_ × $\rho_{\omega_{1}\omega_{2}}$.

## Bivariate HO-IRT Analysis Application

### Data and Measures

We focus on the Alcohol Expectancies and Drinking Motives constructs from Project INTEGRATE (Mun et al., 2015) in the current study. Alcohol Expectancies and Drinking Motives are closely associated constructs, although they are different from each other conceptually. Alcohol Expectancies address the positive or negative beliefs about alcohol's cognitive, emotional, and behavioral effects (e.g., It would be easier to express feelings), whereas Drinking Motives address specific reasons for drinking to achieve desired outcomes (e.g., I drink to be sociable). Based on the literature and the preliminary exploratory data analysis, we conceptualized Alcohol Expectancies and Drinking Motives as second-order trait dimensions that are hierarchically and linearly related to the following five first-order trait dimensions: (1) Social Enhancement and Disinhibition Expectancies, (2) Tension Reduction and Relaxation Expectancies, (3) Negative Alcohol Expectancies, (4) Coping Motives, and (5) Social and Enhancement Motives. The first three and the latter two first-order dimensions represent Alcohol Expectancies and Drinking Motives, respectively.

The entire item data pool consisted of a total of 126 items assessed in 19 studies from several questionnaires and items. These items were originally from the Comprehensive Effects of Alcohol Questionnaire (CEOA; Fromme et al., 1993), the revised Alcohol Expectancies Questionnaire (AEQ; George et al., 1995), the Sex-Related Alcohol Expectancy Questionnaire (SRAEQ; Dermen and Cooper, 1994), the Expectancy/Context Questionnaire (ECQ; Darkes and Goldman, 1993, 1998), and the Drinking Motives Questionnaire Revised (DMQ-R; Cooper, 1994). For some of the questionnaires, the responses were coded identically across studies and, for others, responses were obtained using different response formats. Therefore, the different polytomous response options were converted to binary responses to harmonize item responses across studies, a standard strategy in the field (e.g., Curran et al., 2008, 2014; Bauer, 2017).

Analyzing the entire data set in a single analysis was challenging because the combined data were very sparse (i.e., a high rate of missing data at the item level due to different study designs), which is typical in the analysis of existing IPD from multiple studies. We followed the same strategies used in Huo et al. (2015) to address this challenge. Briefly, we increased common linkage among items and reduced the proportion of missingness by identifying pairs of items eligible for item collapsing, and then checked these collapsed items more closely during the item calibration stage. For the purpose of illustration, we chose a subset of the data with fairly good item coverage (93%) across three studies in terms of data completeness. Most of the missing data within this subset of data occurred at the participant level; not at the group (study) level. Because the participants had a subset of the item response data, we assumed that any missing items in the data were missing at random, and that any inference bias may be ignorable. Accordingly, we could reasonably expect to obtain stable estimates. The data analyzed contained a total of 49 items from three studies (studies 6, 15, and 16) out of the full data set. The total combined sample size was 665 (*N* = 115, 263, and 287, respectively). There were 15 items on dimension 1; 6 items on dimension 2; 13 items on dimension 3; 5 items on dimension 4; and 10 items on dimension 5.

### Comparison of the Results From Univariate and Bivariate HO-IRT Models

We analyzed the data using four different HO-IRT models: univariate HO-IRT model with a common correlation structure across groups (model 1); univariate HO-IRT model with different correlation/covariance structures across groups (model 2); bivariate HO-IRT model with a common correlation structure across groups (model 3); and bivariate HO-IRT model with different correlation/covariance structures across groups (model 4). In each model estimation, Group 1 was set as the anchor group because it had moderate mean responses on average, compared to the other two groups. A total of four chains with different starting values were implemented to monitor convergence. Each chain had 75,000 draws and the initial 10,000 iterations were considered as the burn-in. The G-R statistics (Gelman and Rubin, 1992) for all parameters were <1.1, indicating that convergence was reached for all models. To assess the extent to which the samples drawn from a given chain were independent, the effective sample sizes (ESS) were computed for all parameters of model 4. The ESS ranged from 1535.93 to 6573.40 across the four chains, with an average ESS of 2684.11.

We compared the model fit of the four models based on the deviance information criterion (DIC; Spiegelhalter et al., 2002). DIC is well-suited to compare models that were estimated from the MCMC analysis, and can be treated as a Bayesian extension of the Akaike information criterion (AIC; Akaike, 1974), with lower scores indicating better fit. To compute DIC statistics, half of the deviance variance was used as the effective number of parameters (Gelman et al., 2004). The DIC values for models 1 through 4 were 35571, 32133, 35379, and 31976, respectively. These results indicated that bivariate HO-IRT models (models 3 and 4) outperformed their univariate counterparts (models 1 and 2). In addition, the models with different correlation/covariance structures (models 2 and 4) showed a much better model fit than the models with a common correlation structure across groups (models 1 and 3). Both univariate and bivariate HO-IRT models with different correlation/covariance matrices across groups (models 2 and 4) showed noticeable between-group differences, suggesting that the HO-IRT models with a common correlation matrix were not ideal for the data. Overall, model 4 was the best fitting model. Because the DIC values of models 2 and 4 were relatively close, we report results from models 2 and 4 in Table 5.

**Table 5:** Derived correlation matrices from the bivariate HO-IRT model (lower off-diagonal) and from the univariate HO-IRT model (upper off-diagonal).

		**θ_1_**	**θ_2_**	**θ_3_**	**θ_4_**	**θ_5_**
	θ_1_	1	0.813	0.847	0.442	0.364
	θ_2_	0.800	1	0.827	0.431	0.356
Group 1	θ_3_	0.829	0.809	1	0.450	0.371
	θ_4_	0.447	0.436	0.452	1	0.193
	θ_5_	0.372	0.362	0.375	0.219	1
	θ_1_	1	0.783	0.645	0.615	0.506
	θ_2_	0.792	1	0.646	0.616	0.507
Group 2	θ_3_	0.677	0.674	1	0.507	0.417
	θ_4_	0.482	0.480	0.410	1	0.398
	θ_5_	0.455	0.454	0.387	0.708	1
	θ_1_	1	0.864	0.859	0.737	0.685
	θ_2_	0.866	1	0.836	0.718	0.667
Group 3	θ_3_	0.862	0.840	1	0.713	0.663
	θ_4_	0.723	0.705	0.702	1	0.569
	θ_5_	0.689	0.671	0.668	0.725	1

*In the bivariate HO-IRT model, the first three first-order trait scores (θ_1_, θ_2_, θ_3_) share the same second-order trait ω_1_ and the remaining two first-order trait scores θ_4_ and θ_5_ share ω_2_. Underlined numbers indicate the higher-order relationships. Group-specific correlations between the two second-order latent traits were 0.961, 0.624, and 0.879, respectively for Groups 1–3*.

Table 5 shows three sets of correlation matrices among five first-order latent traits for the three groups for the bivariate HO-IRT model (lower off-diagonal; model 4) and for the univariate HO-IRT model (upper off-diagonal; model 2). In both HO-IRT models, the correlations of the first three first-order trait dimensions were strong in all three groups. However, the correlations of the last two first-order trait dimensions varied across groups and across the two models. In Group 1, the correlations from both HO-IRT models were rather weak (0.219 and 0.193 for models 4 and 2, respectively). However, in Group 2, they were 0.708 vs. 0.398 for models 4 and 2, respectively; in Group 3, they were 0.725 vs. 0.569 for models 4 and 2, respectively. The results suggest that the bivariate HO-IRT model better captured the expected relationships between the first-order latent traits. In contrast, the univariate HO-IRT model did not show evidence of a two-cluster hierarchical data structure. This difference between the two HO-IRT models can be attributed to different formulations of the second-order latent traits in these models. The formulation of the two dimensions at the second-order latent trait level in the bivariate HO-IRT model provided greater flexibility in capturing the associations between dimensions 4 and 5 at the first-order latent trait level for Groups 2 and 3. Furthermore, the bivariate HO-IRT model yielded group-specific correlations between the two second-order latent trait levels: 0.961, 0.624, and 0.879, respectively, for the three groups. When the correlation between the two second-order latent traits was high (i.e., 0.961 for Group 1), both the bivariate and univariate HO-IRT models produced similar correlation/covariance matrices. However, when the correlation was moderate (i.e., 0.624 for Group 2), the covariance matrices for the first-order trait dimensions from the two HO-IRT models diverged.

Finally, we implemented the posterior predictive model check (PPMC) procedure to evaluate the model fit. We used the proportion correct (i.e., the proportion of items endorsing the “correct,” “agree” or “true” response) as a discrepancy measure for this procedure. The posterior predictive *p*-values (ppp; Meng, 1994; Sinharay et al., 2006) for all items from the best fitting model (model 4) fell between 0.025 and 0.975, suggesting that the model was reasonable for the data.

## Discussion

The current study provides findings from a simulation study as well as from real data analysis, which demonstrates the feasibility of the MCMC algorithms and potential utility of the multivariate HO-IRT model for multiple groups in connection with analysis of IPD from multiple studies. In recent years, a number of flexible IRT and FA programs have emerged for estimating unidimensional or multidimentional 1PL, 2PL, 3PL, graded response, partial credit, higher-order IRT, and bifactor models, including BMIRT II, a component of a Bayesian multivariate IRT (BMIRT) toolkit by Yao (2010); IRTPRO (Cai et al., 2011); exMIRT (Cai, 2013); FLIRT (Jeon et al., 2014); a general-purpose IRT program (Haberman, 2013); Mplus (with Bayes estimator; Muthén and Muthén, 1998-2018); Stan (Bürkner, 2017); or WinBUGS (Lunn et al., 2000).

Despite the advances, there is an unmet need for additional tools to help address the challenges of analyzing item response data to combine and synthesize IPD from multiple studies. The existing approaches to establishing commensurate scores across studies have been limited to unidimensional and first-order item response data. For example, a two-parameter logistic IRT (2PL-IRT) model (Curran et al., 2008), a moderated non-linear factor analysis model (MNLFA; Bauer and Hussong, 2009; Curran et al., 2014; Witkiewitz et al., 2016; Bauer, 2017; Hussong et al., 2019), and a longitudinal invariant Rasch test (LIRT; McArdle et al., 2009) are measurement models for unidimensional or multi-unidimensional constructs without any second-order constructs. The 2PL-MUIRT model (Huo et al., 2015) is also unidimensional within each item (i.e., each item measures a single dimension only), except that it can “borrow strength” from related dimensions (i.e., multi-unidimensional) in the pool of items under investigation. Therefore, the development of the proposed multivariate HO-IRT model for multiple groups is a step in the right direction for providing methodological tools to advance the field.

The multivariate HO-IRT model reported in the current study may be appealing as more investigators attempt to combine IPD from different studies and to establish equivalent scores at the participant level for complex item response data. The multivariate HO-IRT model can correctly reflect a theoretical higher-order model for a given construct while estimating latent traits at different hierarchical levels across studies, providing greater flexibility. We demonstrated that the MCMC algorithm accurately estimated the item parameters, and first-order and second-order latent trait scores, as well as the parameters of the hierarchical structures (i.e., the means and regression coefficients) in the simulation study. From the real data application, we found that a bivariate HO-IRT model with different correlation/covariance structures for studies fit the data best as expected, compared with its counterpart univariate HO-IRT model or with the bivariate HO-IRT model with unreasonable constraints (i.e., the same means and covariances across studies).

Note that although we used a multi-group approach to reflect study-level differences, other approaches also exist, such as adding individual-level and study-by-individual level covariates into measurement models when deriving commensurate scores as in MNLFA (Bauer and Hussong, 2009; Curran et al., 2014, 2016). The multiple-indicator and multiple-cause model (MIMIC) approach also accounts for the influence of both categorical (e.g., study membership) and numerical covariates when estimating individual scores. However, results from both of the MNLFA and MIMIC approaches can be quite challenging to interpret (see Lee et al., 2013; Bauer, 2017). In the absence of individual participant data, others have attempted to combine aggregate data, such as correlation/covariance matrices, from multiple studies (e.g., meta-analytic structural equation modeling, Cheung, 2014, 2015). More studies are needed to develop new approaches and compare their relative performance.

The multivariate HO-IRT model and the MCMC algorithms in the present study were developed to address the measurement and computational challenges in the original project. However, the algorithms can be adapted to accommodate new features and converted into program codes (e.g., Stan; Bürkner, 2017) and developed further into a package for R (R Core Team, 2018) in future studies for better accessibility.

With regard to specific areas for model refinement in future studies, first, the current study assumed that the means of the higher-order latent traits are 0 s and their variances are 1 s for model simplicity. Setting such model constraints is not the only solution to handle estimation indeterminacy. Depending on specific research requirements, different constraints can be imposed. In the future, different mean levels of higher-order latent traits, not necessarily 0 s, may be estimated to better understand how higher-order structural parameters function in multiple-group applications. Similarly, for even more complex situations, such as third-order latent traits subsuming second-order latent traits, more constraints may be considered to model data parsimoniously.

Second, we assumed that the same items administered in different studies had the same item parameters. This is the same assumption made for the hierarchical 2PL-MUIRT model (Huo et al., 2015). All participants in the data application were college students from the same college campus who were assessed at the same time or a year apart from one another, and their demographic and alcohol-related characteristics were generally similar. In addition, in an earlier larger study from Project INTEGRATE, we found that there was no meaningful difference between the model constrained to have the same item parameters across studies and the model that allowed items to have different item parameters (DIF items; see Mun et al., 2015 for detailed discussion on the relationship between DIF and latent traits). Therefore, the assumption that item parameters are the same across studies may be reasonable for this study. In future studies, it may be beneficial to further refine model parameterization and estimation to test which of the items may function differently in a large sample with limited missing data.

Finally, the simulation study was rather limited in scope. We focused on demonstrating the feasibility of the MCMC algorithms. It would be helpful to examine several key data conditions under which the MCMC algorithms perform well in a carefully designed simulation study. In sum, the multivariate HO-IRT model for multiple groups has room to further improve.

Having described the areas to improve, we now highlight the promise of this new method in broader terms. In the field of clinical research, it is increasingly important to share and link data across different systems measured in different time scales for data-driven discoveries to deliver faster and more individualized treatment decisions (i.e., the Precision Medicine Initiative; Collins and Varmus, 2015). The National Institutes of Health has long promoted the use of standard measures of phenotypes, such as the PhenX toolkit (https://www.phenxtoolkit.org/), in health research. When measures are not the same across studies, the current paper shows how to establish a “common metric” for complex item response data. With new measurement models specifically developed for such applications, it would be feasible to validly assign psychometrically comparable scale scores to individuals from different studies despite using different questions or surveys, as long as at least some of the items across studies can properly be linked. The new multivariate HO-IRT model for multiple groups that we developed and tested in the current paper may be instrumental for appropriately linking complex item responses given by individual participants from multiple studies to examine them as a whole and expedite scientific discoveries (Hesse et al., 2015; Goldstein and Avenevoli, 2018).

## Ethics Statement

The North Texas Regional IRB reviewed and approved this study.

## Author Contributions

E-YM developed ideas and secured funding for the parent project, oversaw the entire analytical plan, and drafted the manuscript. YH developed the MCMC, analyzed data, drafted technical sections, and contributed to the writing of the manuscript. HRW contributed to the preparation of real data for analysis and edited the manuscript. SS edited the manuscript. JdlT developed and oversaw the analytical plan for this paper and contributed to the writing of this manuscript.

### Conflict of Interest Statement

The authors declare that the research was conducted in the absence of any commercial or financial relationships that could be construed as a potential conflict of interest.

## References

[B1] Akaike H. (1974). A new look at the statistical model identification. IEEE Trans. Autom. Control 19, 716–723. 10.1109/TAC.1974.1100705

[B2] Azevedo C. L. N. Fox J.-P. Andrade D. F. (2015). Longitudinal multiple-group IRT modelling: covariance pattern selection using MCMC and RJMCMC. Int. J. Quant. Res. Educ. 2, 213–243. 10.1504/IJQRE.2015.071737

[B3] Bacci S. Bartolucci F. Gnaldi M. (2014). A class of multidimensional latent class IRT models for ordinal polytomous item responses. Commun. Stat. 43, 787–800. 10.1080/03610926.2013.827718

[B4] Bartolucci F. (2007). A class of multidimensional IRT models for testing unidimensionality and clustering items. Psychometrika 72, 141–157. 10.1007/s11336-005-1376-9

[B5] Bartolucci F. Bacci S. Gnaldi M. (2015). Statistical Analysis of Questionnaires: A Unified Approach Based on Stata and R. New York, NY: CRC Press 10.1201/b18735

[B6] Bauer D. J. (2017). A more general model for testing measurement invariance and differential item functioning. Psychol. Methods 22, 507–526. 10.1037/met0000077 27266798 PMC5140785

[B7] Bauer D. J. Hussong A. M. (2009). Psychometric approaches for developing commensurate measures across independent studies: traditional and new models. Psychol. Methods 14, 101–125. 10.1037/a0015583 19485624 PMC2780030

[B8] Brincks A. Montag S. Howe G. W. Huang S. Siddique J. Ahn S.. (2018). Addressing methdologic challenges and minimizing threats to validity in synthesizing findings from individual-level data across longitudinal randomized trials. Prevent. Sci. 19, 60–73. 10.1007/s11121-017-0769-1 28434055 PMC5651214

[B9] Bürkner P.-C. (2017). brms: an R package for Bayesian multilevel models using Stan. J. Stat. Softw. 80, 1–28. 10.18637/jss.v080.i01

[B10] Cai L. (2013). flexMIRT Version 2: Flexible Multilevel Multidimensional Item Analysis and Test Scoring [Computer software]. Chapel Hill, NC: Vector Psychometric Group.

[B11] Cai L. du Toit S. H. C. Thissen D. (2011). IRTPRO: Flexible, Multidimensional, Multiple Categorical IRT Modeling [Computer software]. Lincolnwodd, IL: Scientific Software International.

[B12] Camilli G. Fox J.-P. (2015). An aggregate IRT procedure for exploratory factor analysis. J. Educ. Behav. Stat. 40, 377–401. 10.3102/1076998615589185

[B13] Casella G. George E. I. (1992). Explaining the Gibbs sampler. Am. Stat. 46, 167–174. 10.1080/00031305.1992.10475878

[B14] Cheung M. W.-L. (2014). Fixed-and random-effects meta-analytic structural equation modeling: examples and analyses in R. Behav. Res. Methods 46, 29–40. 10.3758/s13428-013-0361-y 23807765

[B15] Cheung M. W.-L. (2015). Meta-Analysis: A Structural Equation Modeling Approach. New York, NY: Wiley 10.1002/9781118957813

[B16] Chib S. Greenberg E. (1995). Understanding the Metropolis-Hastings algorithm. Am. Stat. 49, 327–335. 10.1080/00031305.1995.10476177

[B17] Cho S.-J. Cohen A. S. (2010). A multilevel mixture IRT model with an application to DIF. J. Educ. Behav. Stat. 35, 336–370. 10.3102/1076998609353111

[B18] Choi S. Schalet B. Cook K. Cella D. (2014). Establishing a common metric for depressive symptoms: linking the BDI-II, CES-D, and PHQ-9 to PROMIS depression. Psychol. Assess. 26, 513–527. 10.1037/a0035768 24548149 PMC5515387

[B19] Collins F. S. Varmus H. (2015). A new initiative on precision medicine. N. Engl. J. Med. 372, 793–795. 10.1056/NEJMp1500523 25635347 PMC5101938

[B20] Cooper M. L. (1994). Motivations for alcohol use among adolescents: development and validation of a four-factor model. Psychol. Assess. 6, 117–128. 10.1037/1040-3590.6.2.117

[B21] Curran P. J. Cole V. Bauer D. J. Hussong A. M. Gottfredson N. (2016). Improving factor score estimation through the use of observed background characteristics. Struct. Equat. Model. Multidisc. J. 23, 827–844. 10.1080/10705511.2016.1220839 28757790 PMC5526637

[B22] Curran P. J. Cole V. Giordano M. Georgeson A. R. Hussong A. M. Bauer D. J. (2017). Advancing the study of adolescent substance use through the use of integrative data analysis. Eval. Health Prof. 41, 216–245. 10.1177/0163278717747947 29254369 PMC6637746

[B23] Curran P. J. Hussong A. M. (2009). Integrative data analysis: the simultaneous analysis of multiple data sets. Psychol. Methods 14, 81–100. 10.1037/a0015914 19485623 PMC2777640

[B24] Curran P. J. Hussong A. M. Cai L. Huang W. Chassin L. Sher K. J.. (2008). Pooling data from multiple longitudinal studies: the role of item response theory in integrative data analysis. Dev. Psychol. 44, 365–380. 10.1037/0012-1649.44.2.365 18331129 PMC2894156

[B25] Curran P. J. McGinley J. S. Bauer D. J. Hussong A. M. Burns A. Chassin L.. (2014). A moderated nonlinear factor model for the development of commensurate measures in integrative data analysis. Multivariate Behav. Res. 49, 214–231. 10.1080/00273171.2014.889594 25960575 PMC4423418

[B26] Darkes J. Goldman M. S. (1993). Expectancy challenge and drinking reduction: experimental evidence for a mediational process. J. Consult. Clin. Psychol. 61, 344–353. 10.1037/0022-006X.61.2.344 8473588

[B27] Darkes J. Goldman M. S. (1998). Expectancy challenge and drinking reduction: process and structure in the alcohol expectancy network. Exp. Clin. Psychopharmacol. 6, 64–76. 10.1037/1064-1297.6.1.64 9526147

[B28] de la Torre J. Hong Y. (2010). Parameter estimation with small sample size: a higher-order IRT model approach. Appl. Psychol. Meas. 34, 267–285. 10.1177/0146621608329501

[B29] de la Torre J. Patz R. J. (2005). Making the most of what we have: a practical application of multidimensional item response theory in test scoring. J. Educ. Behav. Stat. 30, 295–311. 10.3102/10769986030003295

[B30] de la Torre J. Song H. (2009). Simultaneous estimation of overall and domain abilities: a higher-order IRT model approach. Appl. Psychol. Meas. 33, 620–639. 10.1177/0146621608326423

[B31] Dermen K. H. Cooper M. L. (1994). Sex-related alcohol expectancies among adolescents: I. Scale development. Psychol. Addict. Behav. 8, 152–160. 10.1037/0893-164X.8.3.152

[B32] Doornik J. A. (2009). Object-Oriented Matrix Programming Using Ox (Version 3.1) [Computer software]. London: Timberlake Consultants Press.

[B33] Embretson S. Reise S. (2000). Item Response Theory for Psychologists. Mahwah, NJ: Erlbaum.

[B34] Fox J.-P. Glas C. A. W. (2001). Bayesian estimation of a multilevel IRT model using Gibbs sampling. Psychometrika 66, 271–288. 10.1007/BF02294839

[B35] Fox J.-P. Marianti S. (2016). Joint modeling of ability and differential speed using responses and response times. Multivariate Behav. Res. 51, 540–553. 10.1080/00273171.2016.1171128 27269482

[B36] Fried E. I. Flake J. K. (2018). Measurement matters. Observer 31, 29–31. Available online at: https://www.psychologicalscience.org/observer/measurement-matters

[B37] Fromme K. Stroot E. A. Kaplan D. (1993). Comprehensive effects of alcohol: development and psychometric assessment of a new expectancy questionnaire. Psychol. Assess. 5, 19–26. 10.1037/1040-3590.5.1.19

[B38] Gelman A. Carlin J. B. Stern H. S. Rubin D. B. (2004). Bayesian Data Analysis, 2nd Edn. Boca Raton, FL: Chapman & Hall/CRC.

[B39] Gelman A. Rubin D. B. (1992). Inference from iterative simulation using multiple sequences. Stat. Sci. 7, 457–472. 10.1214/ss/1177011136

[B40] George W. H. Frone M. R. Cooper M. L. Russell M. Skinner J. B. Windle M. (1995). A revised alcohol expectancy questionnaire: factor structure confirmation and invariance in a general population sample. J. Stud. Alcohol 56, 177–185. 10.15288/jsa.1995.56.177 7760564

[B41] Gibbons R. D. Hedeker D. (1992). Full information item bi-factor analysis. Psychometrika 57, 423–436. 10.1007/BF02295430

[B42] Gibbons R. D. Weiss D. J. Frank E. Kupfer D. (2016). Computerized adaptive diagnosis and testing of mental health disorders. Annu. Rev. Clin. Psychol. 12, 83–104. 10.1146/annurev-clinpsy-021815-093634 26651865

[B43] Gnaldi M. Bacci S. Bartolucci F. (2016). A multilevel finite mixture item response model to cluster examinees and schools. Adv. Data Anal. Classif. 10, 53–70. 10.1007/s11634-014-0196-0

[B44] Goldstein A. B. Avenevoli S. (2018). Strength in numbers. Prevent. Sci. 19, 109–111. 10.1007/s11121-017-0856-3 29222616

[B45] Haberman S. J. (2013). A General Program for Item-Response Analysis That Employs the Stabilized Newton-Raphson Algorithm. (ETS Research Rep. No. RR-13-32). Princeton, NJ: Educational Testing Service 10.1002/j.2333-8504.2013.tb02339.x

[B46] Hambleton R. K. Swaminathan H. (1985). Item Response Theory: Principles and Applications. Boston, MA: Kluwer-Nijhoff 10.1007/978-94-017-1988-9

[B47] Hedges L. Olkin I. (1985). Statistical Methods for Meta-Analysis. New York, NY: Academic Press.

[B48] Hesse B. W. Moser R. P. Riley W. T. (2015). From Big Data to Knowledge in the social sciences. Ann. Am. Acad. Pol. Soc. Sci. 659, 16–32. 10.1177/0002716215570007 26294799 PMC4539961

[B49] Huang H.-Y. Wang W.-C. (2013). Higher-order testlet response models for hierarchical latent traits and testlet-based items. Educ. Psychol. Meas. 73, 491–511. 10.1177/0013164412454431

[B50] Huang H.-Y. Wang W.-C. (2014). Multilevel higher-order item response theory models. Educ. Psychol. Meas. 74, 495–515. 10.1177/0013164413509628

[B51] Huang H.-Y. Wang W.-C. Chen P.-H. Su C.-M. (2013). Higher-order item response models for hierarchical latent traits. Appl. Psychol. Meas. 37, 619–637. 10.1177/0146621613488819

[B52] Huo Y. de la Torre J. Mun E.-Y. Kim S.-Y. Ray A. E. Jiao Y.. (2015). A hierarchical multi-unidimensional IRT approach for analyzing sparse, multi-group data for integrative data analysis. Psychometrika 80, 834–855. 10.1007/s11336-014-9420-2 25265910 PMC4379139

[B53] Hussong A. M. Curran P. J. Bauer D. J. (2013). Integrative data analysis in clinical psychology research. Annu. Rev. Clin. Psychol. 9, 61–89. 10.1146/annurev-clinpsy-050212-185522 23394226 PMC3924786

[B54] Hussong A. M. Gottfredson N. C. Bauer D. J. Curran P. J. Haroon M. Chandler R.. (2019). Approaches for creating comparable measures of alcohol use symptoms: harmonization with eight studies of criminal justice populations. Drug Alcohol Depend. 194, 59–68. 10.1016/j.drugalcdep.2018.10.003 30412898 PMC6312501

[B55] Jeon M. Rijmen F. Rabe-Hesketh S. (2014). Flexible item response theory modeling with FLIRT. Appl. Psychol. Meas. 38, 404–405. 10.1177/0146621614524982

[B56] Klein Entink R. H. Fox J.-P. van der Linden W. J. (2009). A multivariate multilevel approach to the modelling of accuracy and speed of test takers. Psychometrika 74, 21–48. 10.1007/s11336-008-9075-y 20037635 PMC2792348

[B57] Lee N. Cadogan J. W. Chamberlain L. (2013). The MIMIC model and formative variables: problems and solutions. AMS Rev. 3, 3–17. 10.1007/s13162-013-0033-1

[B58] Liu X. (2008). Parameter expansion for sampling a correlation matrix: an efficient GPX-RPMH algorithm. J. Stat. Comput. Simul. 78, 1065–1076. 10.1080/00949650701519635

[B59] Liu X. Daniels M. J. (2006). A new efficient algorithm for sampling a correlation matrix based on parameter expansion and re-parameterization. J. Comput. Graph. Stat. 15, 897–914. 10.1198/106186006X160681

[B60] Lunn D. J. Thomas A. Best N. Spiegelhalter D. (2000). WinBUGS – A Bayesian modelling framework: concepts, structure, and extensibility. Stat. Comput. 10, 325–337. 10.1023/A:1008929526011

[B61] McArdle J. J. Grimm K. J. Hamagami F. Bowles R. P. Meredith W. (2009). Modeling life-span growth curves of cognition using longitudinal data with multiple samples and changing scales of measurement. Psychol. Methods 14, 126–149. 10.1037/a0015857 19485625 PMC2831479

[B62] Meng X. L. (1994). Posterior predictive p-values. Ann. Stat. 22, 1142–1160. 10.1214/aos/1176325622

[B63] Mun E.-Y. de la Torre J. Atkins D. C. White H. R. Ray A. E. Kim S.-Y.. (2015). Project INTEGRATE: an integrative study of brief alcohol interventions for college students. Psychol. Addict. Behav. 29, 34–48. 10.1037/adb0000047 25546144 PMC4388772

[B64] Mun E.-Y. Ray A. E. (2018). Integrative data analysis from a unifying research synthesis perspective, in Alcohol Use Disorders: A Developmental Science Approach to Etiology, eds Fitzgerald H. E. Puttler L. I. (New York, NY: Oxford University Press), 341–353. 10.1093/oso/9780190676001.003.0020

[B65] Muthén L. Muthén B. (1998-2018). Mplus: Statistical Analysis With Latent Variables (Version 8.2) [Computer software]. Los Angeles, CA: Muthén & Muthén.

[B66] R Core Team (2018). R: A Language and Environment for Statistical Computing. Vienna: R Foundation for Statistical Computing. Retrieved from: http://www.R-project.org/

[B67] Rijmen F. Jeon M. von Davier M. Rabe-Hesketh S. (2014). A third order item response theory model for modeling the effects of domains and subdomains in large-scale educational assessment surveys. J. Educ. Behav. Stat. 39, 235–256. 10.3102/1076998614531045

[B68] Schmidt S. Zlatkin-Troitschanskaia O. Fox J. P. (2016). Pretest-posttest-posttest multilevel IRT modeling of competence growth of students in higher education in Germany. J. Educ. Measure. 53, 332–351. 10.1111/jedm.12115

[B69] Sheng Y. Wikle C. K. (2008). Bayesian multidimensional IRT models with a hierarchical structure. Educ. Psychol. Meas. 68, 413–430. 10.1177/0013164407308512

[B70] Siddique J. de Chavez P. J. Howe G. Cruden G. Brown C. H. (2018). Limitations in using multiple imputation to harmonize individual participant data for meta-analysis. Prevent. Sci. 19, 95–108. 10.1007/s11121-017-0760-x 28243827 PMC5572105

[B71] Sinharay S. Johnson M. S. Stern H. S. (2006). Posterior predictive assessment of item response theory models. Appl. Psychol. Meas. 30, 298–321. 10.1177/0146621605285517

[B72] Spiegelhalter D. J. Best N. G. Carlin B. P. van der Linde A. (2002). Bayesian measures of model complexity and fit (with discussion). J. R. Stat. Soc. Ser. B 64, 583–639. 10.1111/1467-9868.00353

[B73] Streiner D. L. Norman G. R. Cairney J. (2015). Health Measurement Scales: A Practical Guide to Their Development and Use, 5th Edn New York, NY: Oxford University Press 10.1093/med/9780199685219.001.0001

[B74] van der Linden W. J. (2007). A hierarchical framework for modeling speed and accuracy on test items. Psychometrika 72, 287–308. 10.1007/s11336-006-1478-z

[B75] Van der Linden W. J. Hambleton R. K. (1997). Handbook of Modern Test Theory. New York, NY: Springer 10.1007/978-1-4757-2691-6

[B76] Witkiewitz K. Hallgren K. A. O'Sickey A. J. Roos C. R. Maisto S. A. (2016). Reproducibility and differential item functioning of the alcohol dependence syndrome construct across four alcohol treatment studies: an integrative data analysis. Drug Alcohol Depend. 158, 86–93. 10.1016/j.drugalcdep.2015.11.001 26613839 PMC4698096

[B77] Yao L. (2010). BMIRT: Bayesian Multivariate Item Response Theory (Version 2) [Computer software]. Monterey, CA. Available online at: www.BMIRT.com

