# Supplementary material for: Multivariate Higher-Order IRT Model and MCMC Algorithm for Linking Individual Participant Data From Multiple Studies
Source: Front Psychol. 2019 Jun 12;10:1328. doi: 10.3389/fpsyg.2019.01328 (PMC6582193; doi:10.3389/fpsyg.2019.01328)
Supplement: Supplementary file 1 [file Data_Sheet_1.docx]

**Appendix**

Below we outline the steps for the MCMC algorithms, in particular, how parameters can be sampled from their full conditional distributions. At iteration $t$,

1. For $\boldsymbol{\theta}_{i(g)}$, draw the candidate values $\theta_{i(g)(d)}^{*}$ from $N\left( \theta_{i\left( g \right)\left( d \right)}^{(t-1)}, \sigma_{\theta}^{2} \right)$, where $\sigma_{\theta}^{2}$ is the common candidate variance of $\theta_{i(g)(d)}$, and accept $\boldsymbol{\theta}_{i(g)}^{*}$, with probability

$$\min\left\{ 1,\frac{P\left( \boldsymbol{\theta}_{i\left( g \right)}^{\left( * \right)}|\boldsymbol{\omega}_{i\left( g \right)}^{\left( t-1 \right)}, \boldsymbol{\mu}_{\left( g \right)}^{\left( t-1 \right)}, {\boldsymbol{\sigma}^{2}}_{\left( g \right)}^{\left( t-1 \right)}, \boldsymbol{\lambda}_{\left( g \right)}^{\left( t-1 \right)} \right)L\left( \boldsymbol{X}_{\left( g \right)}|\boldsymbol{\theta}_{i\left( g \right)}^{*}, \boldsymbol{\alpha}^{\left( t-1 \right)}, \boldsymbol{\beta}^{(t-1)} \right)}{P\left( \boldsymbol{\theta}_{i\left( g \right)}^{\left( t-1 \right)}|\boldsymbol{\omega}_{i\left( g \right)}^{\left( t-1 \right)}, \boldsymbol{\mu}_{\left( g \right)}^{\left( t-1 \right)}, {\boldsymbol{\sigma}^{2}}_{\left( g \right)}^{\left( t-1 \right)}, \boldsymbol{\lambda}_{\left( g \right)}^{\left( t-1 \right)} \right)L\left( \boldsymbol{X}_{\left( g \right)}|\boldsymbol{\theta}_{i\left( g \right)}^{(t-1)}, \boldsymbol{\alpha}^{\left( t-1 \right)}, \boldsymbol{\beta}^{(t-1)} \right)} \right\}.$$

2. Draw $\boldsymbol{\Sigma}_{\boldsymbol{\omega}_{\left( g \right)}}$ from the Inverse-Wishart distribution,

$$\boldsymbol{\Sigma}_{\boldsymbol{\omega}_{\left( g \right)}}\sim Inverse Wishart\left( \nu_{I}=\nu_{0}+I, \Lambda_{I}^{-1}=\Lambda_{0}+\Sigma\boldsymbol{\omega}\boldsymbol{\omega}^{'} \right).$$

The sampled $\boldsymbol{\Sigma}_{\boldsymbol{\omega}_{\left( g \right)}}$ is then transformed into the corresponding $\boldsymbol{\rho}_{\boldsymbol{\omega}_{\left( g \right)}}$. The provisional $\boldsymbol{\rho}_{\boldsymbol{\omega}_{\left( g \right)}}$ is accepted based on the M-H acceptance probability set by comparing the determinants of the provisional and previous values, that is,

$$\min\left\{ 1,\frac{\left| \boldsymbol{\rho}_{\boldsymbol{\omega}_{\left( g \right)}}^{(*)} \right|}{\left| \boldsymbol{\rho}_{\boldsymbol{\omega}_{\left( g \right)}}^{(t-1)} \right|} \right\}.$$

3. For $\boldsymbol{\omega}_{i(g)}$, draw the candidate values $\omega_{i(g)(d)}^{*}$ from $N\left( \omega_{i\left( g \right)\left( d \right)}^{(t-1)}, \sigma_{\omega}^{2} \right)$, where $\sigma_{\omega}^{2}$ is the common candidate variance of $\omega_{i(g)(d)}$, and accept $\boldsymbol{\omega}_{i(g)}^{*}$, with probability

$$\min\left\{ 1,\frac{P\left( \boldsymbol{\omega}_{i\left( g \right)}^{*} \right)P\left( \boldsymbol{\theta}_{i\left( g \right)}^{\left( t \right)}|\boldsymbol{\omega}_{i\left( g \right)}^{*}, \boldsymbol{\mu}_{\left( g \right)}^{\left( t-1 \right)}, {\boldsymbol{\sigma}^{2}}_{\left( g \right)}^{\left( t-1 \right)}, \boldsymbol{\lambda}_{\left( g \right)}^{\left( t-1 \right)} \right)}{P\left( \boldsymbol{\omega}_{i\left( g \right)}^{(t-1)} \right)P\left( \boldsymbol{\theta}_{i\left( g \right)}^{\left( t \right)}|\boldsymbol{\omega}_{i\left( g \right)}^{(t-1)}, \boldsymbol{\mu}_{\left( g \right)}^{\left( t-1 \right)}, {\boldsymbol{\sigma}^{2}}_{\left( g \right)}^{\left( t-1 \right)}, \boldsymbol{\lambda}_{\left( g \right)}^{\left( t-1 \right)} \right)} \right\}.$$

4. For $\boldsymbol{\lambda}_{(g)}$, draw the candidate values $\lambda_{(g)(d)}^{*}$ from $N\left( \lambda_{\left( g \right)\left( d \right)}^{(t-1)}, \sigma_{\lambda}^{2} \right)$, where $\sigma_{\lambda}^{2}$ is the common candidate variance of $\lambda_{(g)(d)}$, and accept $\lambda_{(g)(d)}^{*}$, with probability

$$\min\left\{ 1,\frac{P\left( \lambda_{(g)(d)}^{*} \right)P\left( \theta_{i\left( g \right)(d)}^{\left( t \right)}|\omega_{i\left( g \right)(d)}^{(t)}, \mu_{\left( g \right)(d)}^{\left( t-1 \right)}, {\sigma^{2}}_{\left( g \right)(d)}^{\left( t-1 \right)}, \lambda_{\left( g \right)(d)}^{*} \right)}{P\left( \lambda_{(g)(d)}^{(t-1)} \right)P\left( \theta_{i\left( g \right)(d)}^{\left( t \right)}|\omega_{i\left( g \right)(d)}^{(t)}, \mu_{\left( g \right)(d)}^{\left( t-1 \right)}, {\sigma^{2}}_{\left( g \right)(d)}^{\left( t-1 \right)}, \lambda_{\left( g \right)(d)}^{(t-1)} \right)} \right\}.$$

5. For ${\boldsymbol{\sigma}^{\mathbf{2}}}_{(g)}$, draw the candidate values ${\sigma^{2}}_{(g)(d)}^{*}$ from $N\left( {\sigma^{2}}_{\left( g \right)\left( d \right)}^{(t-1)}, \sigma_{\sigma^{2}}^{2} \right)$, where $\sigma_{\sigma^{2}}^{2}$ is the common candidate variance of ${\sigma^{2}}_{(g)(d)}$, and accept ${\sigma^{2}}_{(g)(d)}^{*}$, with probability

$$\min\left\{ 1,\frac{P\left( {\sigma^{2}}_{(g)(d)}^{*} \right)P\left( \theta_{i\left( g \right)(d)}^{\left( t \right)}|\omega_{i\left( g \right)(d)}^{(t)}, \mu_{\left( g \right)(d)}^{\left( t-1 \right)}, {\sigma^{2}}_{\left( g \right)(d)}^{*}, \lambda_{\left( g \right)(d)}^{(t)} \right)}{P\left( {\sigma^{2}}_{(g)(d)}^{(t-1)} \right)P\left( \theta_{i\left( g \right)(d)}^{\left( t \right)}|\omega_{i\left( g \right)(d)}^{(t)}, \mu_{\left( g \right)(d)}^{\left( t-1 \right)}, {\sigma^{2}}_{\left( g \right)(d)}^{\left( t-1 \right)}, \lambda_{\left( g \right)(d)}^{(t)} \right)} \right\}.$$

6. Draw the components of $\boldsymbol{\mu}_{g}$ from the multivariate normal distribution expressed above. Alternatively, elements of $\boldsymbol{\mu}_{(g)}$ can be drawn from multiple univariate normal distributions, respectively, if the conditional $\boldsymbol{\theta}_{(g)}$ are used, because as stated above, the dimensions of the conditional $\boldsymbol{\theta}_{(g)}$ are mutually independent.

7. For $\alpha_{j(d)}$ and $\beta_{j(d)}$, draw the candidate values $\alpha_{j(d)}^{*}$ and $\beta_{j(d)}^{*}$from $N\left( \alpha_{j(d)}^{(t-1)}, \sigma_{\alpha}^{2} \right)$ and $N\left( \beta_{j(d)}^{(t-1)}, \sigma_{\beta}^{2} \right)$, respectively, where $\sigma_{\alpha}^{2}$ and $\sigma_{\beta}^{2}$ are the common candidate variances of $\alpha_{j(d)}$ and $\beta_{j(d)}$, respectively, and accept $\alpha_{j(d)}^{*}$ and $\beta_{j(d)}^{*}$, with probabilities

$$\min\left\{ 1,\frac{P\left( \alpha_{j(d)}^{*} \right)L\left( \boldsymbol{X}_{\left( d \right)}|\boldsymbol{\theta}_{\left( g \right)}^{(t)}, \alpha_{j(d)}^{*}, \beta_{j(d)}^{(t)} \right)}{P\left( \alpha_{j(d)}^{(t-1)} \right)L\left( \boldsymbol{X}_{\left( d \right)}|\boldsymbol{\theta}_{\left( g \right)}^{t}, \alpha_{j(d)}^{(t)}, \beta_{j(d)}^{(t-1)} \right)} \right\},$$

and

$$\min\left\{ 1,\frac{P\left( \beta_{j(d)}^{*} \right)L\left( \boldsymbol{X}_{\left( d \right)}|\boldsymbol{\theta}_{\left( g \right)}^{(t)}, \alpha_{j(d)}^{(t)}, \beta_{j(d)}^{*} \right)}{P\left( \beta_{j(d)}^{(t-1)} \right)L\left( \boldsymbol{X}_{\left( d \right)}|\boldsymbol{\theta}_{\left( g \right)}^{t}, \alpha_{j(d)}^{(t-1)}, \beta_{j(d)}^{(t)} \right)} \right\}.$$

Multiple chains (e.g., four chains) with different initial values can be run to monitor the convergence of the algorithm. Each chain can have $m$ iterations, and the first $n$ iterations of each chain can be discarded as the burn-in. The convergence for the structural parameters (i.e., the item parameters, mean matrix, and covariance matrix) can be determined using the Gelman-Rubin (G-R; Gelman & Rubin, 1992) statistics. The G-R diagnostic statistics compare the ratio of the weighted average of the within-chain variance and between-chain variance to the within-chain variance. If this ratio is close to 1 (e.g., less than 1.1 or 1.2; Gelman et al., 2004), it indicates that the chains have reached the stationary distribution.

The estimates of interest are based on the posterior mean (i.e., expected a posteriori; EAP), and are computed as $\hat{\Phi}=\frac{1}{m-n}\sum_{t=n+1}^{m} \Phi^{(t)}$, where $\Phi$ can be any of the following: $\boldsymbol{\theta}_{\left( g \right)}, \boldsymbol{\omega}_{\left( \boldsymbol{g} \right)}, \boldsymbol{\mu}_{\left( \boldsymbol{g} \right)}, {\boldsymbol{\sigma}^{\mathbf{2}}}_{\left( \boldsymbol{g} \right)}, \boldsymbol{\lambda}_{\left( \boldsymbol{g} \right)}, \boldsymbol{\alpha}, \boldsymbol{\beta}$. To fully utilize the results obtained from multiple chains with different starting values, the formula shown above is calculated and averaged across multiple chains when deriving final estimates.
